# The influence of gingival phenotype on the morphology of the maxillary central papilla

**DOI:** 10.1186/s12903-021-01400-x

**Published:** 2021-01-23

**Authors:** Šimon Belák, Radovan Žižka, Martin Starosta, Jana Zapletalová, Jiří Šedý, Michal Štefanatný

**Affiliations:** 1 Department of Periodontology and Oral Medicine, Institute of Dentistry and Oral Sciences, Palacký University, Palackého 12, Olomouc, 779 00 Czech Republic; 2 Czech Educational and Dental Research Innovative Group (CEDRIG), Brno, Czech Republic; 3 Department of Medical Biophysics, Faculty of Medicine and Dentistry, Palacký University, Hnevotínská 3, Olomouc, 775 15 Czech Republic; 4 Department of Anatomy, Faculty of Medicine and Dentistry, Palacký University, Olomouc, Czech Republic

**Keywords:** Human, Gingival phenotype, Gingival thickness, Papilla height, Papilla width, Papilla fill

## Abstract

**Background:**

Preservation of the interdental papilla is an essential part of the functional and esthetic rehabilitation of dental treatment. It has been described that thicker gingival tissues are more resistant to recession. The main objective of this investigation was to analyze whether a thin gingival phenotype represents a potential risk indicator affecting interdental papilla fill, height, or width in an esthetic region between maxillary central incisors. The secondary goals were: (1) to analyze parameters describing the papilla—fill, height, width, and effect of papilla base width on the vertical papillary dimension; (2) to determine correlation between different non-invasive measurements of gingival thickness; (3) to compare both sexes.

**Methods:**

A total of 54 periodontally healthy students (20–30 years old) were included in the study. Gingival thickness was measured using Pirop Ultrasonic Biometer. Gingival phenotype was also assessed by gingival probe transparency. Papilla height and width were measured, and the degree of papilla recession was classified.

**Results:**

No significant relationship between papilla fill, height, width and gingival probe transparency or gingival thickness was found. Gingival thickness and gingival probe transparency showed a significant relationship (P < 0.001). There was a significant relationship between papilla height and papilla fill (P = 0.028). A papilla which filled the interdental space completely seemed to be shorter. A strong positive correlation between papilla height and papilla width was found (P < 0.0001). The papilla between maxillary central incisors was significantly higher in males (P = 0.01).

**Conclusion:**

The appearance of the interdental papilla may be influenced by various factors. Within the limitations of this study, the results showed that the thin gingival phenotype alone is no potential risk indicator affecting interdental papilla fill, height, or width. It seems that there may be some effect of papilla base width on its vertical dimension. Gingival probe transparency is a simple reliable method of assessment of gingival thickness with a threshold value of 1-mm gingival thickness between the thick and thin phenotypes.

## Background

The patient’s demands on dental treatment are often very high. Patients do not seek treatment only for functional rehabilitation, but also for a natural esthetic result. A balanced size, shape, position, and color of teeth are essential components of a successful esthetic outcome and should be in harmony with the surrounding soft tissues [1]. However, in some situations, specific problems with the so-called pink esthetic occur, comprising the mucogingival conditions, such as excessive gingival display, uneven gingival contours, exposure of root surfaces, or loss of the interdental papilla. In such cases, it is also essential to analyze the patient’s smile line [2].

In exposed regions, the interdental papilla plays a vital role in the final esthetic outcome, especially if a high smile line is present. Physiologically, the interdental papilla in the anterior region has a pyramidal shape and fills the entire space under the contact point between two adjacent teeth. If a papilla does not fill the whole interdental space, a black triangle occurs. It is considered an esthetic impairment, and it can also cause phonetic problems or food retention, which can adversely affect periodontal health [3]. Thus, clinicians should be able to adequately analyze the factors related to the interproximal papilla to prevent its loss.

Various situations can influence the morphology of the interdental papilla, particularly periodontal attachment loss resulting in the recession and impairment of the volume of the alveolar bone relative to the interproximal contact [3]. Tarnow et al. observed that critical distance from the contact point/area to the alveolar bone crest was 5 mm [4]. Other investigations also revealed a significant correlation between an increasing interdental distance and papilla recession [5–9]. Soft tissue thickness in relation to the interdental papilla has been investigated in very few studies. There is an assumption that thicker gingival tissues are more resistant to physical trauma and have a lower risk of recession due to the better blood supply and adequate amount of dense fibrous tissue [10]. Periodontal phenotype is determined by gingival phenotype defined as three-dimensional volume of the gingiva and by bone morphotype (thickness of the buccal bone plate) [11]. There is evidence reporting a correlation between gingival thickness and buccal bone plate [12].

Some authors found that a thick gingival phenotype was observed with significantly greater papillary fill [13, 14], but with decreased papillary height [15, 16]. On the other hand, opposite results have been published, wherein a thin phenotype was associated with a significantly higher presence of complete papilla fill [17]. Only one recent study has found papilla width to be an independent predictive factor of periodontal biotype [18]. Many other investigations revealed no statistically significant correlation between the gingival phenotype and the morphology of the interdental papilla [1, 19, 20].

To date, it is not clear whether the gingival phenotype represents a significant factor associated with the morphology of the interdental papilla and whether there are any differences between both sexes. The main objective of this investigation was to analyze wheather a thin gingival phenotype represents a potential risk indicator affecting interdental papilla fill, height, or width in an esthetic region between maxillary central incisors. The secondary goals were: (1) to analyze parameters describing the papilla and the effect of papilla base width on the vertical papillary dimension; (2) to determine the correlation between different non-invasive measurements of gingival thickness; (3) to compare both sexes. We decided to include only maxillary central incisors as the reference area because differences between phenotypes are the most explicit for these teeth and because this region is the most exposed part of the dental arch, posing a major challenge in terms of esthetics [21–23].

## Methods

### Participants

All clinical measurements were performed between April 2017 and August 2017 in the Department of Periodontology and Oral Medicine at the Institute of Dentistry and Oral Sciences in Olomouc, Czech Republic. A total of 57 undergraduate students of dentistry (32 females, 25 males; age range 20–30 years) were enrolled in this study. All participants were thoroughly educated in the field of oral hygiene. All subjects were required to have state of *Gingival health* on an intact periodontium according to a new classification scheme [24]: no clinical attachment level loss; probing pocket depth (assuming no pseudo pockets) ≤ 3 mm; and no bleeding on probing and no obvious gingival color pigmentation at examined sites [25]. The exclusion criteria were as follows:

1. medication intake or suffering from any disorder classified at *Systemic diseases and conditions that affect the periodontal supporting tissues* [11];
2. pregnant or lactating females;
3. heavy smokers (10 and more cigarettes per day);
4. lack of keratinized tissue width (≤ 2 mm) in the region of upper central incisors;
5. tooth position anomalies in the region of maxillary central incisors;
6. any restorations in the area of maxillary central incisors.

Three subjects were excluded because of insufficient data obtained due to their failure to attend the second appointment.

### Data collection

All measurements were performed by one experienced and previously calibrated examiner (Š.B.). Intra-examiner reproducibility was achieved by reassessing 20 random subjects to find the accuracy between repeated measurements.

**Fig. 1:**
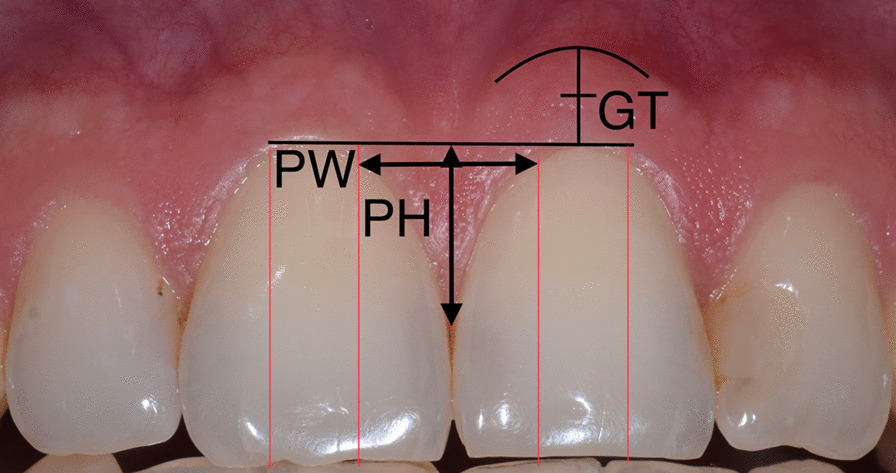
Reference point for ultrasonic measurement of gingival thickness (GT), papilla height (PH), and papilla width (PW)

At the first appointment, gingival thickness was measured using a non-invasive Pirop Ultrasonic Biometer (Echo-Son, Krancowa, Poland) with the A-scan probe (tip diameter 1.7 mm) with 20 MHz frequency and 1540 m/s ultrasonic impulse velocity and an accuracy of up to 0.01 mm. A chlorhexidine gel was applied to the tip of the probe, which was gently applied to the reference point on the intersection between the mid-facial longitudinal axis of the left upper central incisor and the horizontal axis of the keratinized mucosa at the midpoint of mucogingival and free gingival grove (Fig. 1). Each assessment was based on ten automatic measurements. These were averaged and displayed on the screen of the device. The standard deviation of the mean value from ten automatic measurements did not exceed 0.05 mm.

**Fig. 2:**
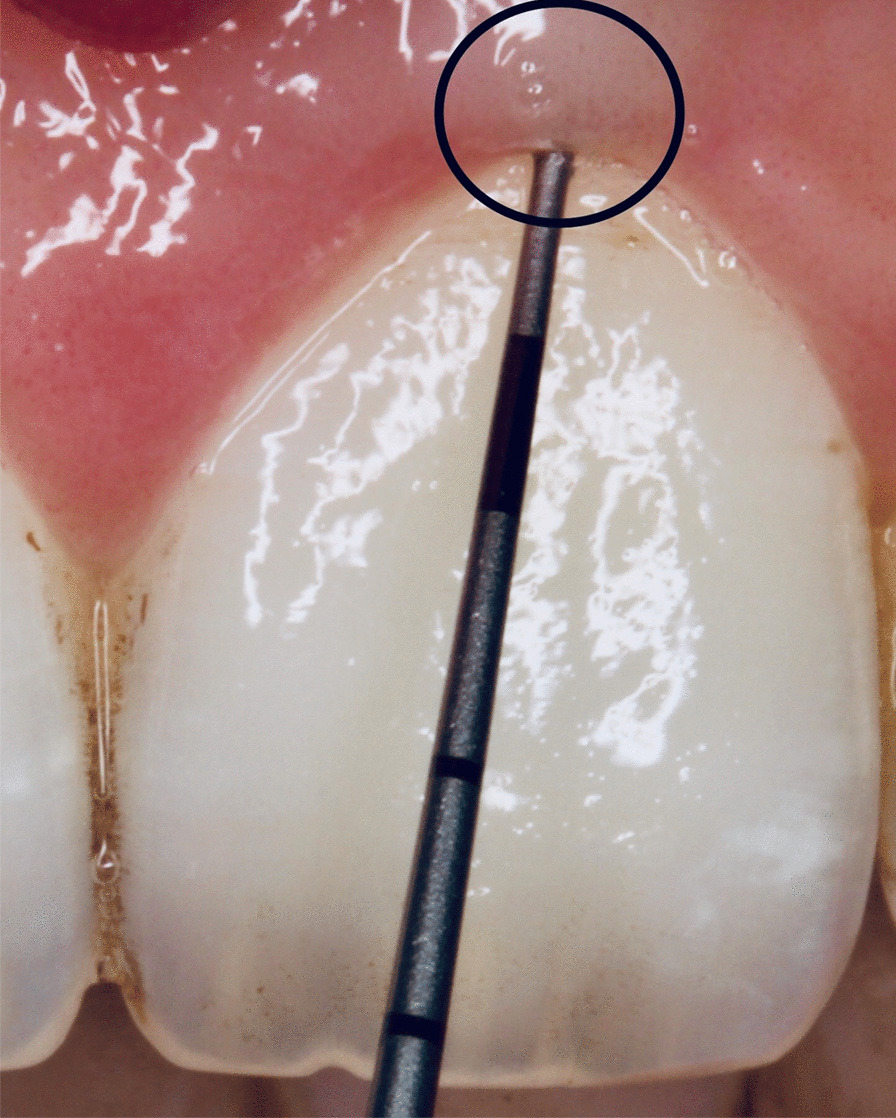
Gingival probe transparency: thick gingival phenotype

**Fig. 3:**
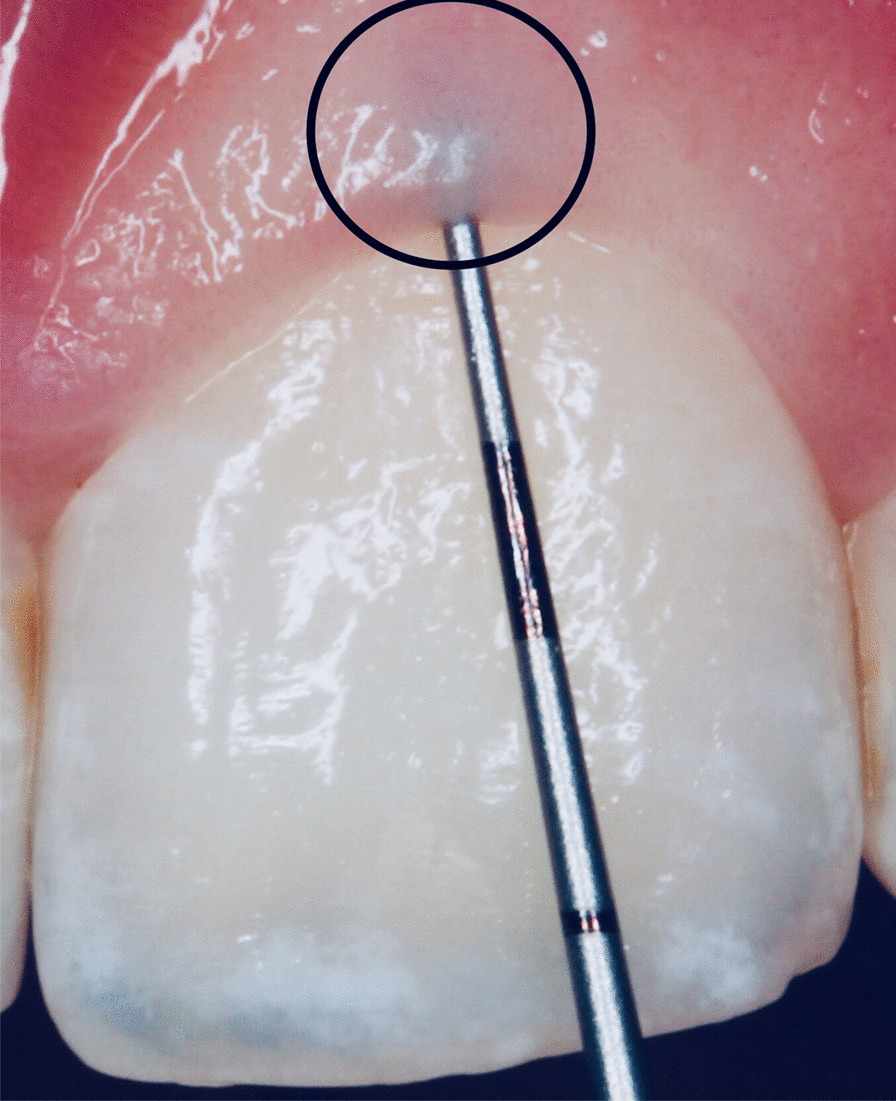
Gingival probe transparency: thin gingival phenotype

At the next appointment scheduled a month later, an intraoral photograph of the anterior maxillary region was taken in a standardized manner—unified shooting conditions and camera setting parameters. A pressure-sensitive periodontal probe, with a controlled (~ 0.25 N) force to the apical end (Carl Martin 973/SP, Solingen, Germany), was placed in the center of the facial aspect of the gingival sulcus of the left maxillary central incisor to assess gingival probe transparency [26]. If the periodontal probe was visible through the gingival sulcus, the phenotype was categorized as thin. If the periodontal probe was not visible, the phenotype was assessed as thick (Figs. 2 and 3).

**Fig. 4:**
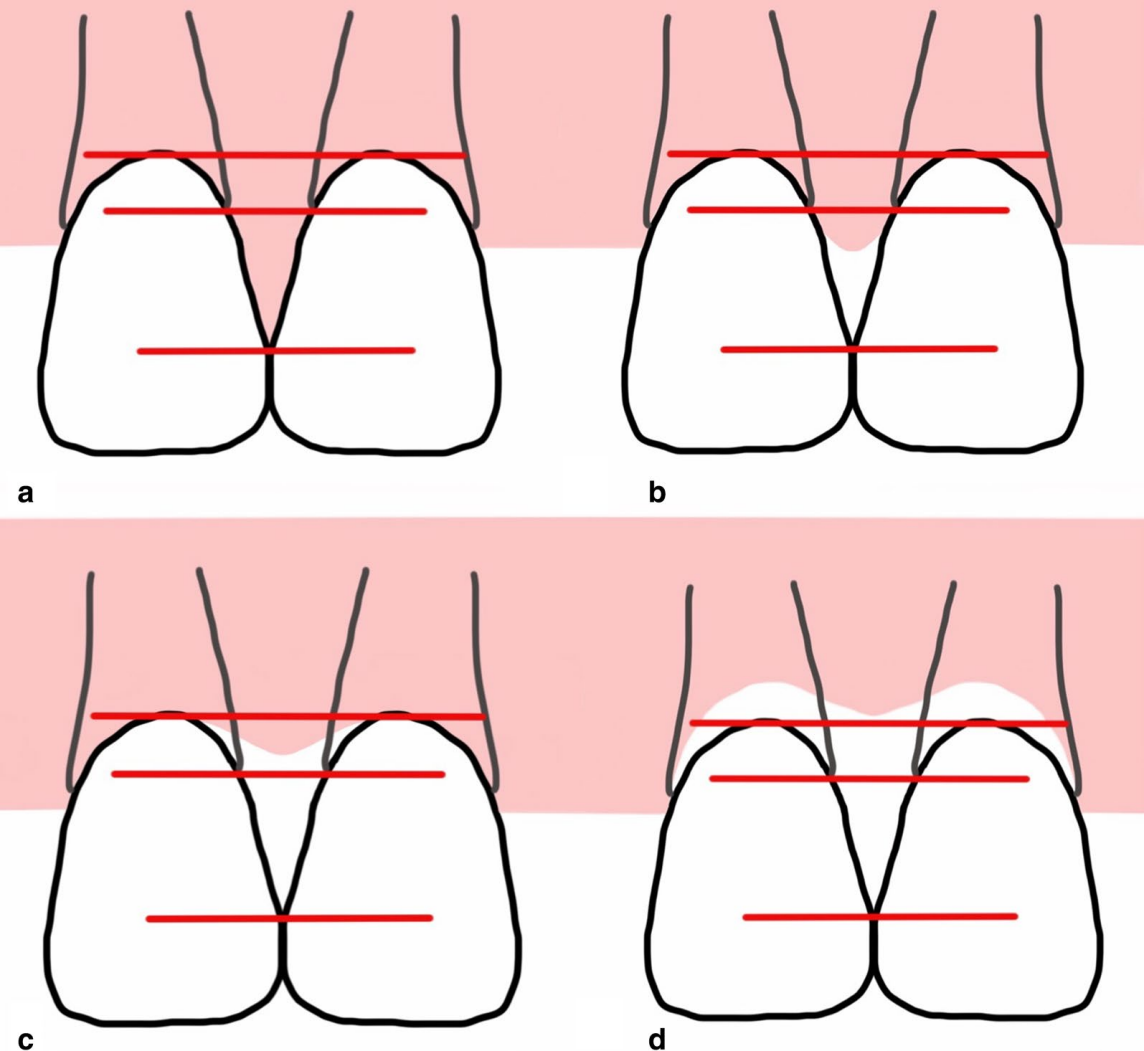
The assessment of papilla fill proposed by Nordland and Tarnow [27]: **a** Normal, **b** Class 1, **c** Class 2, **d** Class 3

Papilla fill between maxillary central incisors was assessed from the photographs using the classification proposed by Nordland and Tarnow [27]. It is based on three anatomical landmarks: the coronal part of the proximal cementoenamel junction (CEJ), the apical extent of the facial CEJ, and interdental contact point/area. The classification is as follows: (a) Normal—the papilla fills the entire interdental space up to the contact point/area; (b) Class 1—the tip of the interdental papilla is located between the contact point/area and the level of the CEJ on the proximal surface of the tooth; (c) Class 2—the tip of the interdental papilla is located on or more apically to the level of the CEJ on the proximal surface of the tooth, but coronally to the level of the facial CEJ; (d) Class 3—the tip of the interdental papilla is located on or apically to the level of the facial CEJ (Fig. 4).

The height and width of the interdental papilla between maxillary central incisors were measured from the intraoral photograph. Image calibration was done using markers from a periodontal probe placed parallelly in the gingival sulcus of the tooth 21, in the Planmeca Romexis dental imaging software (Planmeca, Helsinki, Finland). Papilla height was measured as the distance from the tip of the papilla to the connecting line of the gingival zeniths of maxillary central incisors (Fig. 1). The crown of the central incisor was divided into three equal portions of equal width. The width of the papilla base was measured as the length of the line connecting the level of gingival margins at the border between the mesial and the middle portion of central incisors (Fig. 1).

### Statistical analysis

IBM SPSS Statistics version 22 software was used to analyze the data. Quantitative variables were reported as mean ± standard deviation (SD); categorical variables were reported as absolute and relative frequencies. The correlation between quantitative data was assessed using Spearman’s correlation analysis. The relationship between enumeration data was evaluated using the Chi-square test. The correlation between quantitative and categorical variables was assessed using the Mann–Whitney *U* test. The remeasurements of papilla height and gingival thickness were verified by the Dahlberg formula and the Intraclass correlation coefficient (ICC). Cohen’s kappa coefficient was used to measure intra-rater agreement for the transparency and papilla fill parameters. The normality of the data was verified using the Shapiro–Wilk test. A significance level of less than 0.05 was considered statistically significant.

## Results

The Cohen kappa value showed a substantial agreement (0.61) between original and control measurements of gingival probe transparency. ICC for the gingival thickness parameter (0.933) also showed an excellent match, but the Dahlberg error rate of variation was higher than 5%. The ICC coefficient for papilla height (0.985) and papilla width (0.935) revealed an excellent match; and also, a low Dahlberg error rate indicates a very good match of both measurements. Papilla fill showed absolute agreement in both measurements. There was no statistically significant systematic shift between the measurements.

The age of the participants was 26 ± 1.5 years. Table 1 lists the distribution, mean values, and standard deviation of the clinical data of 54 participants included in this study.

**Table 1:** Distribution of measured parameters

*Parameters*	
Sex	
Female	N = 32 (59.3%)
Male	N = 22 (40.7%)
Papilla fill	
Normal	N = 29 (58.0%)
Class 1	N = 21 (42.0%)
Phenotype (transparency)	
Thick	N = 39 (72.2%)
Thin	N = 15 (27.8%)
Papilla height	4.93 ± 1.06
Papilla width	6.25 ± 0.72
Gingival thickness	0.96 ± 0.25

Data are reported as N (%) or as mean ± standard deviation

Papilla characteristics—height, width and fill in different gingival phenotypes based on gingival probe transparency are shown in Table 2. In the thick phenotype group, papilla recession was seen in 34.3% of cases, while in the thin phenotype group, it was 60%. However, no statistically significant relationship between papilla height and width was confirmed.

**Table 2:** Papilla characteristics—height, width and fill in different gingival phenotypes based on probe transparency

	Thick	Thin	*P *value
Papilla height	4.89 ± 1.07	4,81 ± 1.04	0.505
Papilla width	6.35 ± 0.68	6.0 ± 0.77	0.250
Papilla fill			
Normal	N = 23 (65.7%)	N = 6 (40.0%)	0.091
Class 1	N = 12 (34.3%)	N = 9 (60.0%)	

Data are reported as N (%) or as mean ± standard deviation

Table 3 shows the correlation of gingival thickness with papilla height, width and papilla fill, where no statistically significant relationship was found.

**Table 3:** Correlation of gingival thickness with papilla height, width and papilla fill

	Gingival thickness		*P *value
Papilla height	r	0.097	0.484
Papilla width		0.175	0.205
Papilla fill			
Normal		1.02 ± 0.30	0.238
Class 1		0.901 ± 0.178	

Data are reported as Spearman’s coefficient of rank correlation (r) or as mean ± standard deviation

The relationships between papilla fill and papilla height, width are shown in Table 4. There was a significant relationship between papilla fill and papilla height (P = 0.028). A papilla classified as normal, filling the entire interdental space seemed to be shorter than a Class 1 papilla. No significant correlation between papilla fill and width was found. Spearman’s correlation analysis revealed a strong positive correlation between papilla height and papilla width (r = 0.738, P < 0.0001).

**Table 4:** Relationship between papilla fill and papilla height and width

	Normal	Class 1	*P *value
Papilla height	4.66 ± 1.08	5.35 ± 0.86	0.028
Papilla width	6.17 ± 0.71	6.39 ± 0.79	0.381

Data are reported as mean ± standard deviation

The relationship between gingival probe transparency and gingival thickness (Table 5) was statistically significant (P < 0.001). The mean gingival thickness for the group assessed as thick was greater compared to the thin phenotype group.

**Table 5:** Relationship between gingival probe transparency and gingival thickness

	Thick	Thin	*P *value
Gingival thickness	1.047 ± 0.233	0.748 ± 0.155	< 0.0001

Data are reported as mean ± standard deviation

Differences in measured parameters between sexes were statistically significant for papilla height only (P = 0.01). The mean papilla height was greater in the male group than in the female group. Other parameters revealed no statistical difference (Table 6).

**Table 6:** Sex distribution

	Female	Male	*P *value
Papilla height	4.63 ± 1.02	5.37 ± 0.96	0.010
Papilla width	6.13 ± 0.75	6.43 ± 0.64	0.106
Gingival thickness	0.939 ± 0.290	1.001 ± 0.184	0.137
Papilla fill			
Normal	N = 17 (56.7%)	N = 12 (60.0%)	0.815
Class 1	N = 13 (43.3%)	N = 8 (40.0%)	
Phenotype (transparency)			
Thick	N = 21 (65.6%)	N = 18 (81.8%)	0.192
Thin	N = 11 (34.4%)	N = 4 (18.2%)	

Data are reported as N (%) or as mean ± standard deviation

## Discussion

Preservation of the interdental papilla is an essential part of the functional and esthetic rehabilitation of dental treatment. It has been described that the morphology of the interdental papilla is strongly related to bone volume in the interproximal space [4–9]. In addition to recession of the interdental papilla related to periodontal disease, recession can also occur in a healthy periodontium due to anatomical and physiological predispositions [1]. Therefore, we examined only periodontally healthy patients without any tooth position discrepancies. To have a homogeneous sample, we only studied the papilla between maxillary central incisors, because contact points may vary in different regions, which may influence the shape of the interdental papilla.

Periodontal phenotype is determined by gingival phenotype defined as three-dimensional volume of the gingiva and by bone morphotype (thickness of the buccal bone plate) [11]. There is evidence reporting a correlation between gingival thickness and buccal bone plate [12]; therefore, it was recommended to assess periodontal phenotype in a standardized and reproducible way by an assessment of gingival thickness [11]. In this study, two different non-invasive methods were employed: gingival probe transparency, a method most commonly used in similar studies [16, 19–21, 28, 29], and less standard method of ultrasonic measurement [30–32] of accurate thickness of gingival tissues. A comparison of both methods showed a significant correlation and there seems to be a threshold value of 1-mm gingival thickness between the thick and thin phenotypes assessed by probe transparency, which supports previously published findings by Kan et al. [33]. Therefore, these results support the fact that such a straightforward method of phenotype assessment using gingival probe transparency is as reliable as other methods which are often more time-consuming or require some additional costs for appliances. However, assessment of gingival probe transparency in patients with gingival pigmentation should be made with caution.

Despite the fact that papilla recession was present in 34.3% of cases with the thick phenotype and in 60% of cases with the thin phenotype, there was no statistically significant correlation between the interdental papilla of maxillary central incisors and gingival thickness. This result supports previously published studies by Kim et al. [1] and Singh et al. [19]. Some authors assume that a thick phenotype is more resistant to physical trauma and has a lower risk of papilla recession due to a better blood supply and adequate amount of dense fibrous tissue [34]. The thick phenotype is also associated more strongly with square-shaped tooth crowns with the contact point located more apically, and requires less tissue to fill the interproximal space [22, 23]. This assumption was confirmed by Chow et al. [14] who observed that gingival tissues were significantly thicker when the papilla was competent. Opposite results were published by De Lemos et al. [17] who noted a significantly higher presence of the papilla in the thin phenotype group. In that study, however, the phenotype was evaluated visually only, which may have biased the assessment due to subjectivity. Most of the other authors studied the correlation between phenotype and papilla height as the only descriptive parameter of the papilla. The results suggested that increased papillary height was associated with a thin phenotype [15, 16, 28, 29], which may have been influenced by different tooth shapes [15, 21, 23, 29, 35]. As a tooth becomes triangular, which is more typical for thin phenotype subjects, the contact point can be seen more coronally, and a longer papilla is present. Yin et al. [18] have recently published that papilla width has a significant effect on phenotype, making the papilla of maxillary central incisors of the thin biotype narrower. Our study failed to find an effect on papilla width with gingival thickness. In the study by Yin et al. [18] papilla width was assessed as the distance between the gingival zeniths of two adjacent teeth. The incongruity in measurement methods of papilla width may be a major reason for different results. However, there have been few studies on the correlation between the gingival thickness and papilla width, and more research needs to be done.

We also compared papilla characteristics—papilla fill, height and width between each other. Papilla height was measured as the distance from the tip of the papilla to the connecting line of the gingival zeniths of maxillary central incisors; therefore, it is important to point out, that the values of papilla height do not include complete supracrestal connective tissue attachment (i.e. they do not represent the true height of the papilla). The results showed that a papilla assessed as normal, i.e. one which fills the whole interdental space, seemed to be shorter than that classified as a Class 1, where a slight reduction of papilla fill is present. Chang et al. found in their study that papilla height was significantly greater in the group where a complete papilla was present [36]. However, this result was not confirmed in a study by Kim et al. [37]. Both authors measured papilla height on radiographs using radiopaque material and defined it as the distance from the crest of the bone to the tip of the papilla. It can be speculated that different methods of papilla height measurements may be a major factor contributing to inconsistencies in these results. Another possible factor may be the different ages of the participants, as reported by Chow et al. [14], who noted that papilla height decreased by 0.012 mm with each increasing year of age. In our study, the age of the participants ranged from 20 to 30 years only. We found no significant relationship between papilla fill and papilla width, but a significant correlation was observed between papilla height and width. It seems that there may be an effect of papilla base width on its vertical dimension and therefore may pose as one of the potential risk indicators influencing the presence of interdental papilla.

The mean papilla height was greater in the male group than in the female group. However, the groups were unequal without further statistical correction, the interpretation of the sex comparison should be made with caution. Chow et al. have reported the same results [14], which is in contrast with the study by Joshi et al. [16]. Many other authors have observed a thin phenotype more frequently in females [16, 21, 38]; in this study, however, no correlation was found. We assume that the greater height of the interproximal papilla found in the male group was due to different tooth forms and position of the contact point, which could be the reason for the only difference between sexes in this study.

The small size of our sample lacking different age groups limits the assessment of gingival phenotype and its correlation with papilla morphology. Therefore, in future studies, it is recommended to expand the sample size. To describe gingival phenotype properly, measurement of keratinized tissue width should be considered, although the importance of this parameter is still a matter of discussion. Also, it is advisable to evaluate other potential risk indicators, such as tooth form (shape/dimension, the width of the approximal tooth surfaces, course of the CEJ) or tooth angulation, which appear to be other potential factors influencing the interdental papilla because of a different shape and position of the contact point. Finally, another potential factor, buccolingual tooth position, which may affect the gingival phenotype and thickness of the alveolar bone, should be added in future studies to provide more convincing evidence.

## Conclusion

The appearance of the interdental papilla may be influenced by various factors. Within the limitations of this study, the results showed that the thin gingival phenotype alone is no potential risk indicator affecting interdental papilla fill, height, or width. It seems that there may be some effect of papilla base width on its vertical dimension. Gingival probe transparency is a simple reliable method of assessment of gingival thickness with a threshold value of 1-mm gingival thickness between the thick and thin phenotypes.

## Data Availability

The datasets used and/or analyzed during the current study are available from the corresponding author on reasonable request.

## Abbreviations

PH: Papilla height
PW: Papilla width
GT: Gingival thickness
CEJ: Cementoenamel junction
SD: Standard deviation
ICC: Intraclass correlation coefficient
